# On some generated soft topological spaces and soft homogeneity

**DOI:** 10.1016/j.heliyon.2019.e02061

**Published:** 2019-07-11

**Authors:** Samer Al Ghour, Awatef Bin-Saadon

**Affiliations:** Department of Mathematics and Statistics, Jordan University of Science and Technology, Irbid, Jordan

**Keywords:** Mathematics, Minimal soft open sets, Homogeneous, Generated soft topology

## Abstract

We introduce soft homogeneity as an extension of homogeneity in ordinary topological spaces. Based on the generated soft topology of a given indexed family of classical topologies inspite of a one topology given by Terepeta in [16], we investigate soft minimal open set and homogeneity relation between the generated soft topology and the given indexed family of topologies. We introduce several results, examples and counterexamples.

## 1 Introduction

The classical mathematical theories have difficulties for solving complicated problems which include uncertain data in many areas such as engineering, environment, economics, medical science, social science, etc. Theories of probability, fuzzy sets [1], rough sets [2], intuitionistic fuzzy sets [3] and vague sets [4] are considered as mathematical tools for dealing with uncertainties. Molodtsov [5] justified that each of these theories has its deep-seated difficulty. These difficulties are mainly come from the inadequacy of the parameterization tool of the theories. For dealing with uncertainties away from these difficulties, Molodtsov [5] defined soft sets as follows: Let *X* be an initial universe and *A* be a set of parameters and denote the power set of *X* by P(X), a soft set over *X* relative to *A* is a function F:A⟶P(X). The theory of soft sets has been introduced and studied by several researchers (see [6], [7]). Authors [5], [8] applied soft sets in many areas such as Riemann integration, Perron integration, smoothness of function, operation research, game theory, probability and theory of measurements. Authors [9] applied soft sets in decision-making problems. The notion of soft topological spaces was introduced in [10]. Then, Mathematicians modified several concepts of classical topological spaces to include soft topological spaces (see [11], [12], [13], [14], [15], [16], [17], [18], [19], [20], [21]). A topological space (X,ℑ) is homogeneous if for all x,y∈X, there is a homeomorphism f:(X,ℑ)⟶(X,ℑ) such that f(x)=y. Since homogeneity concepts are of importance in general topology and still a hot area of research, as appears in [22], [23], [24], [25], [26], [27], [28], [29], [30], [31], [32], [33], we see that it is suitable to extend the homogeneity concept to include soft topological spaces. One of our main goals of the present work is to show how the definition of homogeneity in ordinary topological spaces can be modified in order to define its extension in soft topological spaces. This paper is organized as follows.

In section two, we introduce some basic definitions and results which we use them in our research.

In section three, for a given collection $\left\{{ℑ}_{a}:a\in A\right\}$ of topologies on a set *X*, we introduce a generated soft topology *τ* over *X* with a set of parameters *A* such that $\tau_{a}={ℑ}_{a}$ for all a∈A; moreover, for a given topological space (X,ℑ) and a given set of parameters *A*, we show that (X,ℑ) generates a soft topology *τ* on *X* with the set of parameters *A*.

In section four, we continue the study of minimal soft open sets; in particular, we give a mapping regarding minimal soft open sets, also we give a link between the minimal soft open sets of a soft topological space and the minimal open sets of its generated topological spaces and vice versa.

In section five, we introduce and investigate soft homogeneity in soft topological spaces; in particular, we study the relation between soft homogeneity of a soft topological space and some ordinary topological spaces generated by this soft topological space, we study the relation between homogeneity of an ordinary topological space and some soft topological spaces generated by this ordinary topological space, and we introduce some properties of homogeneous soft topological spaces that contains a minimal soft open set.

## 2 Preliminaries

In this section, we introduce some basic definitions and results which we use them in our research.

Definition 2.1 [5] Let *X* be an initial universe and *A* be a set of parameters. A soft set over *X* relative to *A* is a function F:A⟶P(X). The family of all soft sets over *X* relative to *A* will be denoted by SS(X,A).

Definition 2.2 [6] Let F,G∈SS(X,A).

(1) *F* is a soft subset of *G*, denoted by $F\overset{˜}{\subseteq }G$, if F(a)⊆G(a) for each a∈A.

(2) *F* and *G* are said to be soft equal, denoted by F=G if $F\overset{˜}{\subseteq }G$ and $F\overset{˜}{\subseteq }G$.

(3) Union of *F* and *G* is denoted by $F\overset{˜}{\cup }G$ and defined to be the soft set $F\overset{˜}{\cup }G\in SS\left(X,A\right)$ where $\left(F\overset{˜}{\cup }G\right)\left(a\right)=F(a)\cup G\left(a\right)$ for each a∈A.

(4) Intersection of *F* and *G* is denoted by $F\overset{˜}{\cap }G$ and defined to be the soft set $F\overset{˜}{\cap }G\in SS\left(X,A\right)$ where $\left(F\overset{˜}{\cap }G\right)\left(a\right)=F(a)\cap G\left(a\right)$ for each a∈A.

Definition 2.3 [11] Let Δ be an arbitrary index set and $\left\{F_{\alpha }:\alpha \in \Delta \right\}\subseteq SS\left(X,A\right)$.

(a) The union of these soft sets is the soft set denoted by $\overset{˜}{\underset{\alpha \in \Delta }{⋃}}F_{\alpha }$ and defined by $\left(\overset{˜}{\underset{\alpha \in \Delta }{⋃}}F_{\alpha }\right)\left(a\right)=\underset{\alpha \in \Delta }{⋃}F_{\alpha }\left(a\right)$ for each a∈A.

(b) The intersection of these soft sets is the soft set denoted by $\overset{˜}{\underset{\alpha \in \Delta }{⋂}}F_{\alpha }$ and defined by $\left(\overset{˜}{\underset{\alpha \in \Delta }{⋂}}F_{\alpha }\right)\left(a\right)=\underset{\alpha \in \Delta }{⋂}F_{\alpha }\left(a\right)$ for each a∈A.

Definition 2.4 [6] Let F∈SS(X,A).

(a) *F* is called a null soft set over *X* relative to *A*, denoted by $0_{A}$, if F(a)=∅ for each a∈A.

(b) *F* is called an absolute soft set over *X* relative to *A*, denoted by $1_{A}$, if F(a)=X for each a∈A.

Definition 2.5 [12] Let F∈SS(X,A). *F* is called a soft point over *X* relative to *A* if there exist e∈A and x∈X such that

$$F\left(a\right)=\left\{ \begin{array}{cc} \left\{x\right\}\; & \text{if }a=e\\ \emptyset \; & \text{if }a\neq e \end{array}\right.\text{.}$$

We denote *F* by $e_{x}$. The family of all soft points over *X* relative to *A* is denoted by SP(X,A).

Proposition 2.6 *[12]* *Let* $a_{x},b_{y}\in SP\left(X,A\right)$*. Then* $a_{x}=b_{y}$ *iff* x=y *and* a=b*.*

Definition 2.7 [12] Let F∈SS(X,A) and $e_{x}\in SP\left(X,A\right)$. Then $e_{x}$ is said to belong to *F* (notation: $e_{x}\overset{˜}{\in }F$) if $e_{x}\overset{˜}{\subseteq }F$ or equivalently: $e_{x}\overset{˜}{\in }F$ iff x∈F(e).

Proposition 2.8 $F\in SS\left(X,A\right)-\left\{0_{A}\right\}$ *iff there is* $a_{x}\in SP\left(X,A\right)$ *such that* $a_{x}\overset{˜}{\in }F$*.*

Proof Straightforward. □

Proposition 2.9 *[12]* *Let* F,G∈SS(X,A)*. Then the following are equivalent:*

*(a)* $F\overset{˜}{\subseteq }G$*.*

*(b) For all* $a_{x}\in SP\left(X,A\right)$*,* $a_{x}\overset{˜}{\in }F$ *implies* $a_{x}\overset{˜}{\in }G$*.*

Definition 2.10 [12] Let F∈SS(X,A). The set $\left\{a_{x}:a_{x}\overset{˜}{\in }F\right\}$ will be denoted by Pt(F).

It is clear that $SP\left(X,A\right)=Pt\left(1_{A}\right)$.

Proposition 2.11 *[12]* *Let X be an initial universe and A be a set of parameters. Then for any* F,G∈SS(X,A)*,* $F\overset{˜}{\subseteq }G$ *iff* Pt(F)⊆Pt(G)*.*

Definition 2.12 [7] Let SS(X,A) and SS(Y,B) be families of soft sets. Let p:X⟶Y and u:A⟶B be functions. Then a soft mapping $f_{pu}:SS\left(X,A\right)⟶SS\left(Y,B\right)$ is defined as:

(a) Let F∈SS(X,A). The image of *F* under $f_{pu}$, written as $f_{pu}\left(F\right)\in SS\left(Y,B\right)$ is defined by

$$\left(f_{pu}\left(F\right)\right)\left(b\right)=\left\{ \begin{array}{cc} \cup \left\{p\left(F\left(a\right)\right):u\left(a\right)=b\right\}\; & \text{ if }u^{-1}\left(b\right)\neq \emptyset \\ \emptyset \; & \text{if }u^{-1}\left(b\right)=\emptyset \text{.} \end{array}\right.$$

(b) Let G∈SS(Y,B). The inverse of *G* under $f_{pu}$, written as $f_{pu}^{-1}\left(G\right)\in SS\left(X,A\right)$ is defined by

$$\left(f_{pu}^{-1}\left(G\right)\right)\left(a\right)=p^{-1}\left(G\left(u\left(a\right)\right)\right)\text{.}$$

Definition 2.13 [7] Let $f_{pu}:SS\left(X,A\right)⟶SS\left(Y,B\right)$ be a soft mapping. Then $f_{pu}$ is called:

(a) Injective if *p* and *u* are injective.

(b) Surjective if *p* and *u* are surjective.

(c) Bijective if *p* and *u* are bijective.

Proposition 2.14 *Let* $f_{pu}:SS\left(X,A\right)⟶SS\left(Y,B\right)$ *be a soft mapping. Then for all* $a_{x}\in SP\left(X,A\right)$*,* $f_{pu}\left(a_{x}\right)=u{\left(a\right)}_{p\left(x\right)}\in SP\left(Y,B\right)$*.*

Proof Straightforward. □

Proposition 2.15 *Let* $f_{pu}:SS\left(X,A\right)⟶SS\left(Y,B\right)$ *be a soft mapping. Then for all* $a_{x}\in SP\left(X,A\right)$*,* $f_{pu}\left(a_{x}\right)=b_{y}$ *iff* p(x)=y *and* u(a)=b*.*

Proof Straightforward. □

Proposition 2.16 *A soft mapping* $f_{pu}:SS\left(X,A\right)⟶SS\left(Y,B\right)$ *is injective iff* $f_{pu}:SP\left(X,A\right)⟶SP\left(Y,B\right)$ *is injective.*

Proof Straightforward. □

Proposition 2.17 *A soft mapping* $f_{pu}:SS\left(X,A\right)⟶SS\left(Y,B\right)$ *is surjective iff* $f_{pu}:SP\left(X,A\right)⟶SP\left(Y,B\right)$ *is surjective.*

Proof Straightforward. □

Proposition 2.18 *Let* $f_{pu}:SS\left(X,A\right)⟶S\left(Y,B\right)$ *be a soft mapping. Let* $b_{y}\in SP\left(Y,B\right)$ *and* F∈SS(X,A)*. Then* $b_{y}\overset{˜}{\in }f_{pu}\left(F\right)$ *iff there is* $a_{x}\overset{˜}{\in }F$ *such that* $f_{pu}\left(a_{x}\right)=b_{y}$*.*

Proof *Necessity.* Assume that $b_{y}\overset{˜}{\in }f_{pu}\left(F\right)$. Then $y\in \left(f_{pu}\left(F\right)\right)\left(b\right)=\cup \left\{p\left(F\left(a\right)\right):a\in u^{-1}\left(b\right)\right\}$. So, there is $a\in u^{-1}\left(b\right)$ such that y∈p(F(a)). Choose x∈F(a) such that p(x)=y. Thus, we have $a_{x}\overset{˜}{\in }F$ and $f_{pu}\left(a_{x}\right)=u{\left(a\right)}_{p\left(x\right)}=b_{y}$.

*Sufficiency.* Assume that there is $a_{x}\overset{˜}{\in }F$ such that $f_{pu}\left(a_{x}\right)=b_{y}$. Since $b_{y}\overset{˜}{\in }F$, then $f_{pu}\left(a_{x}\right)\overset{˜}{\in }f_{pu}\left(F\right)$ and so $b_{y}\overset{˜}{\in }f_{pu}\left(F\right)$. □

Proposition 2.19 *Let* $f_{pu}:SS\left(X,A\right)⟶SS\left(Y,B\right)$ *be a soft mapping. Let* $a_{x}\in SP\left(X,A\right)$ *and* G∈SS(Y,B)*. Then* $a_{x}\overset{˜}{\in }f_{pu}^{-1}\left(G\right)$ *iff* $f_{pu}\left(a_{x}\right)\overset{˜}{\in }G$*.*

Proof *Necessity.* Assume that $a_{x}\overset{˜}{\in }f_{pu}^{-1}\left(G\right)$. Then $x\in \left(f_{pu}^{-1}\left(G\right)\right)\left(a\right)=p^{-1}\left(G\left(u\left(a\right)\right)\right)$. So, p(x)∈G(u(a)). Thus, $f_{pu}\left(a_{x}\right)=u{\left(a\right)}_{p\left(x\right)}\overset{˜}{\in }G$.

*Sufficiency.* Assume that $f_{pu}\left(a_{x}\right)\overset{˜}{\in }G$. Then $u{\left(a\right)}_{p\left(x\right)}\overset{˜}{\in }G$ and p(x)∈G(u(a)). Thus, $x\in p^{-1}\left(G\left(u\left(a\right)\right)\right)=\left(f_{pu}^{-1}\left(G\right)\right)\left(a\right)$. Hence, $a_{x}\overset{˜}{\in }f_{pu}^{-1}\left(G\right)$. □

Definition 2.20 [10] Let *τ* ⊆ S(X,A). Then *τ* is called a soft topology on *X* relative to *A* if

(1) $0_{A},1_{A}\in \tau$,

(2) the union of any number of soft sets in *τ* belongs to *τ*,

(3) the intersection of any two soft sets in *τ* belongs to *τ*.

The triplet (X,τ,A) is called a soft topological space (STS) over *X* relative to *A*. The members of *τ* are called soft open sets in (X,τ,A) and their complements are called soft closed sets in (X,τ,A).

Definition 2.21 A soft mapping $f_{pu}:\left(X,\tau ,A\right)⟶\left(Y,\sigma ,B\right)$ is called:

(1) Soft continuous [15] if $f_{pu}^{-1}\left(G\right)\in \tau$ for every G∈σ.

(2) Soft open [15] if $f_{pu}\left(F\right)\in \sigma$ for every F∈τ.

(3) A soft homeomorphism [14] if $f_{pu}$ is soft continuous, soft open and bijective.

Definition 2.22 [15] Let $f_{p_{1}u_{1}}:SS\left(X,A\right)⟶SS\left(Y,B\right)$ and $f_{p_{2}u_{2}}:SS\left(Y,B\right)⟶SS\left(Z,C\right)$ be functions, then the composition of $f_{p_{1}u_{1}}$ and $f_{p_{2}u_{2}}$ is soft mapping from SS(X,A) onto SS(Z,C) denoted by $f_{p_{1}u_{1}}\circ f_{p_{2}u_{2}}$ and defined by $f_{p_{1}u_{1}}\circ f_{p_{2}u_{2}}=f_{\left(p_{2}\circ p_{1}\right)\left(u_{2}\circ u_{1}\right)}$.

Proposition 2.23 *[15]* *If* $f_{p_{1}u_{1}}:\left(X,\tau ,A\right)⟶\left(Y,\sigma ,B\right)$ *and* $f_{p_{2}u_{2}}:\left(Y,\sigma ,B\right)⟶\left(Z,\beta ,C\right)$ *are soft continuous, then* $f_{\left(p_{2}\circ p_{1}\right)\left(u_{2}\circ u_{1}\right)}:\left(X,\tau ,A\right)⟶\left(Z,\beta ,C\right)$ *is soft continuous.*

Proposition 2.24 *[13]* *A soft mapping* $f_{pu}:\left(X,\tau ,A\right)⟶\left(Y,\sigma ,B\right)$ *is a soft homeomorphism iff* $f_{pu}$ *is bijective, and* $f_{pu}:\left(X,\tau ,A\right)⟶\left(Y,\sigma ,B\right)$ *and* $f_{p^{-1}u^{-1}}:\left(Y,\sigma ,B\right)⟶\left(X,\tau ,A\right)$ *are soft continuous.*

Proposition 2.25 *Let* (X,τ,A) *be a STS. Then the set of all soft homeomorphisms from* (X,τ,A) *onto* (X,τ,A) *forms a group under the operation* $\left(f_{p_{2}u_{2}}\right)\circ \left(f_{p_{1}u_{1}}\right)=f_{\left(p_{2}\circ p_{1}\right)\left(u_{2}\circ u_{1}\right)}$*.*

Proof Straightforward. □

Proposition 2.26 *[10]* *Let* (X,τ,A) *be a STS. Then the collection* {F(a):F∈τ} *defines a topology on X for every* a∈A*. This topology will be denoted by* $\tau_{a\;}$*.*

Definition 2.27 [28] Let (X,ℑ) be a topological space. A non-empty open subset A⊆X is called a minimal open set if the only open subsets of *A* are *A* and ∅. The set of all minimal open sets of (X,ℑ) will be denoted by min⁡(X,ℑ).

Definition 2.28 [19] Let (X,τ,A) be a soft topological space. A subcollection B of *τ* is called a soft base of *τ* if every member of *τ* can be expressed as a union of members of B.

Proposition 2.29 *[20]* *Let* (X,τ,A) *be a STS and let* B⊆τ*. Then* B *is a soft base for τ if for every* $F\in \tau -\{0_{A}\}$ *and every* $a_{x}\overset{˜}{\in }$ *F, there exists* G∈B *such that* $a_{x}\overset{˜}{\in }G\overset{˜}{\subseteq }$ *F.*

Proposition 2.30 *[17]* *A collection* B⊆SS(X,A) *is a soft base for some soft topology on X relative to A iff the following conditions hold:*

*i.* $\overset{˜}{⋃}\left\{F:F\in B\right\}=1_{A}$*.*

*ii. For every* F,G∈B *and for every* $a_{x}\overset{˜}{\in }F\overset{˜}{\cap }G$*, there is* H∈B *such that* $a_{x}\overset{˜}{\in }H\overset{˜}{\subseteq }F\overset{˜}{\cap }G$*.*

## 3 Soft topologies generated by ordinary topologies

In this section, for a given collection $\left\{{ℑ}_{a}:a\in A\right\}$ of topologies on a set *X*, we introduce a generated soft topology *τ* over *X* with a set of parameters *A* such that $\tau_{a}={ℑ}_{a}$ for all a∈A; moreover, for a given topological space (X,ℑ) and a given set of parameters *A* we show that (X,ℑ) generates a soft topology *τ* on *X* with the set of parameters *A*.

Theorem 3.1 *Let X be an initial universe and let A be a set of parameters. Let* $\left\{{ℑ}_{a}:a\in A\right\}$ *be an indexed family of topologies on X and let*

$$\tau =\left\{F\in SS\left(X,A\right):F\left(a\right)\in {ℑ}_{a}\;\text{for all}\;a\in A\right\}\text{.}$$

Then (X,τ,A) is a STS.

Proof For all a∈A, $\emptyset ,X\in {ℑ}_{a}$ and so $0_{A},1_{A}\in \tau$. Let F,G∈τ, then for all a∈A, $F\left(a\right),G\left(a\right)\in {ℑ}_{a}$ and $F\left(a\right)\cap G\left(a\right)\in {ℑ}_{a}$. So for all a∈A, $F\left(a\right)\cap G\left(a\right)=\left(F\overset{˜}{\cap }G\right)\left(a\right)\in {ℑ}_{a}$ and hence $F\overset{˜}{\cap }G\in \tau$. Let $\left\{F_{\alpha }:\alpha \in \Delta \right\}\subseteq \tau$, then for all a∈A and $\alpha \in \Delta ,F_{\alpha }\left(a\right)\in {ℑ}_{a}$ and $\left(\underset{\alpha \in \Delta }{⋃}F_{\alpha }\left(a\right)\right)\in {ℑ}_{a}$. So for all a∈A, $\left(\underset{\alpha \in \Delta }{⋃}F_{\alpha }\left(a\right)\right)=\left(\overset{˜}{\underset{\alpha \in \Delta }{⋃}}F_{\alpha }\right)\left(a\right)\in {ℑ}_{a}$ and hence $\overset{˜}{\underset{\alpha \in \Delta }{⋃}}F_{\alpha }\in \tau$. □

Definition 3.2 Let *X* be an initial universe and let *A* be a set of parameters. Let $\left\{{ℑ}_{a}:a\in A\right\}$ be an indexed family of topologies on *X*. Then the soft topology $\left\{F\in SS\left(X,A\right):F\left(a\right)\in {ℑ}_{a}\text{ for all }a\in A\right\}$ will be denoted by $\underset{a\in A}{⨁}{ℑ}_{a}$.

For any topological space (X,ℑ) and any set of parameters *A*, denote the family {F∈SS(X,A):F(a)∈ℑ for all a∈A} by τ(ℑ).

Corollary 3.3 *[16]* *For any topological space* (X,ℑ) *and any set of parameters A,* (X,τ(ℑ),A) *is a STS.*

Proof For each a∈A, set ${ℑ}_{a}=ℑ$. Then $\tau \left(ℑ\right)=\underset{a\in A}{⨁}{ℑ}_{a}$ and by Theorem 3.1 we get the result. □

Definition 3.4 Let *X* be an initial universe and let *A* be a set of parameters. For any a∈A and Y⊆X, the soft set F∈SS(X,A) defined by

$$F\left(b\right)=\left\{ \begin{array}{cc} Y\; & \text{if }b=a\\ \emptyset \; & \text{if }b\neq a \end{array}\right.$$

will be denoted by $a_{Y}$.

Theorem 3.5 *Let X be an initial universe and let A be a set of parameters. Let* $\left\{{ℑ}_{a}:a\in A\right\}$ *be an indexed family of topologies on X. Then the family* $\left\{a_{Y}:a\in A\;\text{and}\;Y\in {ℑ}_{a}\right\}$ *is a soft base of* $\underset{a\in A}{⨁}{ℑ}_{a}$*.*

Proof If $F\in \underset{a\in A}{⨁}{ℑ}_{a}-\left\{0_{A}\right\}$, then $F=\overset{˜}{\underset{a\in A}{⋃}}a_{F\left(a\right)}$. □

Corollary 3.6 *Let X be an initial universe and let A be a set of parameters. Let* $\left\{{ℑ}_{a}:a\in A\right\}$ *be an indexed family of topologies on X. If* $B_{a}$ *is a base for* ${ℑ}_{a}$ *for all* a∈A*, then* $\left\{a_{Y}:a\in A\;\text{and}\;Y\in B_{a}\right\}$ *is a soft base of* $\underset{a\in A}{⨁}{ℑ}_{a}$*.*

Theorem 3.7 *Let X be an initial universe and let A be a set of parameters. Let* $\left\{{ℑ}_{a}:a\in A\right\}$ *be an indexed family of topologies on X. Then* ${\left(\underset{a\in A}{⨁}{ℑ}_{a}\right)}_{b}={ℑ}_{b}$ *for all* b∈A*.*

Proof Let $S\in {\left(\underset{a\in A}{⨁}{ℑ}_{a}\right)}_{b}$, then there is $F\in \underset{a\in A}{⨁}{ℑ}_{a}$ such that F(b)=S. By the definition of $\underset{a\in A}{⨁}{ℑ}_{a}$, $F\left(b\right)\in {ℑ}_{b}$ and thus $S\in {ℑ}_{b}$. Conversely, if $S\in {ℑ}_{b}$, then $b_{S}\in \underset{a\in A}{⨁}{ℑ}_{a}$ and so $b_{S}\left(b\right)=S\in {\left(\underset{a\in A}{⨁}{ℑ}_{a}\right)}_{b}$. □

Corollary 3.8 *[16]* *If* (X,ℑ) *is a topological space and A is any set of parameters, then* ${\left(\tau \left(ℑ\right)\right)}_{a}=ℑ$ *for all* a∈A*.*

Proof For each a∈A, set ${ℑ}_{a}=ℑ$. Then $\tau \left(ℑ\right)=\underset{a\in A}{⨁}{ℑ}_{a}$ and by Theorem 3.7 we get the result. □

Theorem 3.9 *If* (X,τ,A) *is a STS, then* $\tau \subseteq \underset{a\in A}{⨁}\tau_{a}$*.*

Proof Let F∈τ, then $F\left(a\right)\in \tau_{a}$ for all a∈A and so $F\in \underset{a\in A}{⨁}\tau_{a}$. □

The following example shows that the equality in Theorem 3.9 does not hold in general:

Example 3.10 Let X={1,2,3,4}, A={a,b}, F={(a,{1,2}),(b,{3,4})}, and $\tau =\left\{0_{A},1_{A},F\right\}$. Then $\tau_{a}=\left\{\emptyset ,X,\left\{1,2\right\}\right\}$, $\tau_{b}=\left\{\emptyset ,X,\left\{3,4\right\}\right\}$ and $\tau_{a}⨁\tau_{b}=\left\{0_{A},1_{A},a_{\left\{1,2\right\}},b_{\left\{3,4\right\}},F\right\}$. So $\tau \neq \tau_{a}⨁\tau_{b}$.

Theorem 3.11 *If* (X,τ,A) *is a STS, then* ${\left(\underset{a\in A}{⨁}\tau_{a}\right)}_{b}=\tau_{b}$ *for all* b∈A*.*

Proof Theorem 3.7. □

Definition 3.12 Let *X* be an initial universe and *A* be a set of parameters. A soft set F∈SS(X,A) such that F(a)=Y for all a∈A will be called a constant soft set and will be denoted by $C_{Y}$. The family of all constant soft sets in SS(X,A) will be denoted by CSS(X,A).

Remark 3.13 Let *X* be an initial universe and *A* be a set of parameters. Then $0_{A}=C_{\emptyset }$ and $1_{A}=C_{X}$.

Lemma 3.14 *Let* $\left\{C_{Y_{\alpha }}:\alpha \in \Delta \right\}\subseteq CSS\left(X,A\right)$*. Then* $\overset{˜}{\underset{\alpha \in \Delta }{⋃}}C_{Y_{\alpha }}=C_{\left(\underset{\alpha \in \Delta }{⋃}Y_{\alpha }\right)}$ *and* $\overset{˜}{\underset{\alpha \in \Delta }{⋂}}C_{Y_{\alpha }}=C_{\left(\underset{\alpha \in \Delta }{⋂}Y_{\alpha }\right)}$*.*

Proof Straightforward. □

Theorem 3.15 *If* (X,ℑ) *is a topological space, then for any set of parameters A, the collection* $\tau =\left\{C_{Y}:Y\in ℑ\right\}$ *is a soft topology on X relative to A.*

Proof Suppose that (X,ℑ) is a topological space. Since ∅, X∈ℑ, then $C_{\emptyset }$, $C_{X}\in \tau$ and so $0_{A}$, $1_{A}\in \tau$. Let $C_{Y}$, $C_{Z}\in \tau$ where Y,Z∈ℑ. Then Y∩Z∈ℑ and so $C_{Y\cap Z}\in \tau$. By Lemma 3.14, $C_{Y}\overset{˜}{\cap }C_{Z}=C_{Y\cap Z}$ and thus $C_{Y}\overset{˜}{\cap }C_{Z}\in \tau$. Let $\left\{\;C_{Y_{\alpha }}:\alpha \in \Delta \right\}\subseteq \tau$ where $\left\{Y_{\alpha }:\alpha \in \Delta \right\}\subseteq ℑ$, then $\underset{\alpha \in \Delta }{⋃}Y_{\alpha }\in ℑ$ and so $C_{\left(\underset{\alpha \in \Delta }{⋃}Y_{\alpha }\right)}\in \tau$. By Lemma 3.14, $\overset{˜}{\underset{\alpha \in \Delta }{⋃}}C_{Y_{\alpha }}=C_{\left(\underset{\alpha \in \Delta }{⋃}Y_{\alpha }\right)}$ and thus $\overset{˜}{\underset{\alpha \in \Delta }{⋃}}C_{Y_{\alpha }}\in ℑ$. This ends the proof that (X,τ,A) is a STS. □

Definition 3.16 If (X,ℑ) is a topological space and *A* is a set of parameters, then we will denote the soft topology $\left\{C_{Y}:Y\in ℑ\right\}$ by C(ℑ).

Theorem 3.17 *If* (X,ℑ) *is a topological space and A is a set of parameters, then* ${\left(C\left(ℑ\right)\right)}_{a}=ℑ$ *for all* a∈A*.*

Proof Clear. □

Theorem 3.18 *Let* (X,τ,A) *be a STS with* τ⊆CSS(X,A)*, then* $\left\{Y\subseteq X:C_{Y}\in \tau \right\}$ *is a topology on X.*

Proof Suppose that (X,τ,A) is a STS with τ⊆CSS(X,A). Let $ℑ=\left\{Y\subseteq X:C_{Y}\in \tau \right\}$. Since $C_{\emptyset }$, $C_{X}\in \tau$, then ∅, X∈ℑ. Let Y,Z∈ℑ. Then $C_{Y}$, $C_{Z}\in \tau$ and so $C_{Y}\overset{˜}{\cap }C_{Z}\in \tau$. By Lemma 3.14, $C_{Y}\overset{˜}{\cap }C_{Z}=C_{Y\cap Z}$. Thus, Y∩Z∈ℑ. Let $\left\{\;Y_{\alpha }:\alpha \in \Delta \right\}\subseteq ℑ$. Then $\left\{C_{Y_{\alpha }}:\alpha \in \Delta \right\}\subseteq \tau$ and so $\overset{˜}{\underset{\alpha \in \Delta }{⋃}}C_{Y_{\alpha }}\in \tau$. By Lemma 3.14, $\overset{˜}{\underset{\alpha \in \Delta }{⋃}}C_{Y_{\alpha }}=C_{\left(\underset{\alpha \in \Delta }{⋃}Y_{\alpha }\right)}$. Thus, $\underset{\alpha \in \Delta }{⋃}Y_{\alpha }\in ℑ$. This ends the proof that (X,ℑ) is a topological space. □

Definition 3.19 If (X,τ,A) is a STS with τ⊆CSS(X,A), then the topology $\left\{Y\subseteq X:C_{Y}\in \tau \right\}$ on *X* will be denoted by D(τ).

Theorem 3.20 *If* (X,τ,A) *is a STS with* τ⊆CSS(X,A)*, then* $\tau_{a}=D\left(\tau \right)$ *for all* a∈A*.*

Remark 3.21 Let (X,ℑ) be a topological space and let *A* be a set of parameters. Then D(C(ℑ))=ℑ.

Remark 3.22 Let (X,τ,A) be a STS with τ⊆CSS(X,A). Then C(D(τ))=τ.

## 4 Minimal soft open sets

In this section, we continue the study of minimal soft open sets; in particular, we give a mapping regarding minimal soft open sets, also we give a link between the soft minimal open sets of a soft topological space and the minimal open sets of its generated topological spaces and vice versa.

Definition 4.1 [21] Let (X,τ,A) be a STS. A soft set F∈SS(X,A) is said to be a minimal soft open set of (X,τ,A) if $F\in \tau -\left\{0_{A}\right\}$ and for all G∈τ with $G\overset{˜}{\subseteq }F$ either $G=0_{A}$ or G=F.

The set of all minimal soft open sets of a STS (X,τ,A) will be denoted by min⁡(X,τ,A).

Proposition 4.2 *[21]* *Let* (X,τ,A) *be a STS. If* F∈min⁡(X,τ,A) *and* G∈τ*, then* $F\overset{˜}{\cap }G=0_{A}$ *or* $F\overset{˜}{\subseteq }G$*.*

Proposition 4.3 *[21]* *Let* (X,τ,A) *be a STS. If* F,G∈min⁡(X,τ,A)*, then* $F\overset{˜}{\cap }G=0_{A}$ *or* F=G*.*

Theorem 4.4 *Let* $f_{pu}:\left(X,\tau ,A\right)⟶\left(Y,\sigma ,B\right)$ *be a soft continuous function. If* F∈min⁡(X,τ,A) *with* $f_{pu}\left(F\right)\in \sigma$*, then* $f_{pu}\left(F\right)\in \min\left(Y,\sigma ,B\right)$*.*

Proof Since F∈min⁡(X,τ,A), then there is $a_{x}\overset{˜}{\in }F$. So, $f_{pu}\left(a_{x}\right)=u{\left(a\right)}_{p\left(x\right)}\overset{˜}{\in }f_{pu}\left(F\right)$ and hence $f_{pu}\left(F\right)\neq 0_{B}$. Suppose $H\in \sigma -\left\{0_{B}\right\}$ with $H\overset{˜}{\subseteq }f_{pu}\left(F\right)$. Choose $b_{y}\overset{˜}{\in }H$. Then $b_{y}\overset{˜}{\in }f_{pu}\left(F\right)$. So, there is $a_{x}\overset{˜}{\in }F$ such that $b_{y}=f_{pu}\left(a_{x}\right)$. Since $f_{pu}$ is soft continuous, then $f_{pu}^{-1}\left(H\right)\in \tau$. Since we have $a_{x}\overset{˜}{\in }F\overset{˜}{\cap }f_{pu}^{-1}\left(H\right)$, then $F\overset{˜}{\cap }f_{pu}^{-1}\left(H\right)\in \tau -\left\{0_{A}\right\}$. So by Proposition 4.2, $F\overset{˜}{\subseteq }f_{pu}^{-1}\left(H\right)$. Hence, $f_{pu}\left(F\right)=f_{pu}\left(f_{pu}^{-1}\left(H\right)\right)\overset{˜}{\subseteq }H$. It follows that $f_{pu}\left(F\right)=H$. Hence, $f_{pu}\left(F\right)\in \min\left(Y,\sigma ,B\right)$. □

Corollary 4.5 *Let* $f_{pu}:\left(X,\tau ,A\right)⟶\left(Y,\sigma ,B\right)$ *be bijective and soft open. If* G∈min⁡(Y,σ,B) *with* $f_{pu}^{{-}^{1}}\left(G\right)\in \tau$*, then* $f_{pu}^{{-}^{1}}\left(G\right)\in \min\left(X,\tau ,A\right)$*.*

Theorem 4.6 *Let* (X,τ,A) *be a STS and let* B *be a soft base for τ. Then* min⁡(X,τ,A)⊆B*.*

Proof Let F∈min⁡(X,τ,A). Choose $a_{x}\overset{˜}{\in }F$. Since B is a soft base for *τ*, then by Proposition 2.29, there exists G∈B such that $a_{x}\overset{˜}{\in }G\overset{˜}{\subseteq }$ *F*. Since F∈min⁡(X,τ,A) and $G\neq 0_{A}$, then F=G. Hence, F∈B. □

Theorem 4.7 *If* F∈min⁡(X,τ,A)*, then for every* a∈A *with* F(a)≠∅*,* $F\left(a\right)\in \min\left(X,\tau_{a}\right)$*.*

Proof Suppose F∈min⁡(X,τ,A) and let a∈A such that F(a)≠∅. Suppose $M\in \tau_{a}-\left\{\emptyset \right\}$ with M⊆F(a). Take G∈τ such that M=G(a). Choose x∈G(a). Then $a_{x}\overset{˜}{\in }G\overset{˜}{\cap }F$. Since F∈min⁡(X,τ,A), then by Proposition 4.2 we have $F\overset{˜}{\subseteq }G$. Therefore, F(a)⊆G(a)=M. Hence, M=F(a). This shows that $F\left(a\right)\in \min\left(X,\tau_{a}\right)$. □

Lemma 4.8 *[16]* *Let* (X,τ,A) *be a STS and let* B *be a soft base for τ. Then for every* a∈A*, the family* {F(a):F∈B} *forms a base for the topology* $\tau_{a}$ *on X.*

The following example shows that the converse of Theorem 4.7 is not true in general:

Example 4.9 Let $X=\{x_{1},x_{2},x_{3},x_{4}\}$ and $A=\{a_{1},a_{2}\}$. Let

$$F_{1}=\left\{\left(a_{1},\{x_{1}\}\right),\left(a_{2},\{x_{1},x_{2}\}\right)\right\},F_{2}=\left\{\left(a_{1},\{x_{2}\}\right),\left(a_{2},\{x_{1},x_{2}\}\right)\right\},F_{3}=\left\{\left(a_{1},\{x_{3}\}\right),\left(a_{2},\{x_{3},x_{4}\}\right)\right\},F_{4}=\left\{\left(a_{1},\{x_{4}\}\right),\left(a_{2},\{x_{3},x_{4}\}\right)\right\},F_{5}=\left\{\left(a_{1},\emptyset \right),\left(a_{2},\{x_{1},x_{2}\}\right)\right\},F_{6}=\left\{\left(a_{1},\emptyset \right),\left(a_{2},\{x_{3},x_{4}\}\right)\right\}.$$

Let $B=\{F_{1},F_{2},F_{3},F_{4},F_{5},F_{6}\}$ and let *τ* be the soft topology on *X* relative to *A* having B as a soft base. For every i=1,2, set $B_{a_{i}}=\left\{F\left(a_{i}\right):F\in B\right\}$. By Lemma 4.8, $B_{a_{1}}=\{\emptyset$, $\left\{x_{1}\},\{x_{2}\},\{x_{3}\},\{x_{4}\}\right\}$ is a base for $\tau_{a_{1}}$ and $B_{a_{2}}=\{\{x_{1},x_{2}\},\{x_{1},x_{2}\}\}$ is a base for $\tau_{a_{2}}$. Now $F_{1}\left(a_{1}\right)\in \min\left(X,\tau_{a_{1}}\right)$ and $F_{1}\left(a_{2}\right)\in \min\left(X,\tau_{a_{2}}\right)$, however, $F_{1}\notin \min\left(X,\tau ,A\right)$ because $F_{5}\in \tau -\left\{0_{A}\right\}$ with $F_{5}\overset{˜}{\subseteq }F_{1}$ but $F_{5}\neq F_{1}$.

Definition 4.10 A collection B⊆SS(X,A) is called a soft partition of $1_{A}$ if the following three conditions hold:

(a) $B\subseteq SS\left(X,A\right)-\left\{0_{A}\right\}$.

(b) If F,G∈B, then $F\overset{˜}{\cap }G=0_{A}$ or F=G.

(c) $\overset{˜}{\cup }\left\{F:F\in B\right\}=1_{A}$.

Theorem 4.11 *For any STS* (X,τ,A)*, the following are equivalent:*

*(a)* $\overset{˜}{\cup }\left\{F:F\in \min\left(X,\tau ,A\right)\right\}=1_{A}$*.*

*(b)* min⁡(X,τ,A) *is a soft partition of* $1_{A}$*.*

*(c)* min⁡(X,τ,A) *is a soft base of* (X,τ,A)*.*

Proof (a) ⟹ (b): By the definition of minimal soft open sets, $\min\left(X,\tau ,A\right)\subseteq SS\left(X,A\right)-\left\{0_{A}\right\}$. Also, by Proposition 4.3 we have $F\overset{˜}{\cap }G=0_{A}$ or F=G for all F,G∈min⁡(X,τ,A). The assumption that $\overset{˜}{\cup }\left\{F:F\in \min\left(X,\tau ,A\right)\right\}=1_{A}$ ends the proof that min⁡(X,τ,A) is a soft partition of $1_{A}$.

(b) ⟹ (c): We apply Proposition 2.29. By the definition of minimal soft open sets we have min⁡(X,τ,A)⊆τ. Let $G\in \tau -\left\{0_{A}\right\}$. Choose $a_{x}\overset{˜}{\in }G$. Then $a_{x}\overset{˜}{\in }1_{A}$. Since, by (b), min⁡(X,τ,A) is a soft partition of $1_{A}$, then $\overset{˜}{\cup }\left\{F:F\in \min\left(X,\tau ,A\right)\right\}=1_{A}$, and so there is F∈min⁡(X,τ,A) such that $a_{x}\overset{˜}{\in }F$. Since $a_{x}\overset{˜}{\in }F\overset{˜}{\cap }G$, then by Proposition 4.2 we have $F\overset{˜}{\subseteq }G$. This shows that min⁡(X,τ,A) is a soft base of (X,τ,A).

(c) ⟹ (a): Obvious. □

Theorem 4.12 *If* B⊆SS(X,A) *is a soft partition of* $1_{A}$*, then for every* a∈A *the set* {F(a):F∈BandF(a)≠∅} *is a soft partition of X.*

Proof Straightforward. □

Theorem 4.13 *Let X be an initial universe and let A be a set of parameters. Let* $\left\{{ℑ}_{a}:a\in A\right\}$ *be an indexed family of topologies on X. Then* $\min\left(X,\underset{a\in A}{⨁}{ℑ}_{a},A\right)=\left\{a_{Y}:a\in A\;\text{and}\;Y\in \min\left(X,{ℑ}_{a}\right)\right\}$*.*

Proof Let $F\in \min\left(X,\underset{a\in A}{⨁}{ℑ}_{a},A\right)$. By Theorems 3.5 and 4.6, there is a∈A and $Y\in {ℑ}_{a}$ such that $F=a_{Y}$. Since $F\in \min\left(X,\underset{a\in A}{⨁}{ℑ}_{a},A\right)$, then it is clear that $Y\in \min\left(X,{ℑ}_{a}\right)$. The other inclusion is straightforward. □

Theorem 4.14 *If* (X,ℑ) *is a topological space and A is a set of parameters, then* $\min\left(X,C\left(ℑ\right),A\right)=\left\{C_{Y}:Y\in \min\left(X,ℑ\right)\right\}$*.*

Proof Let $C_{Y}\in \min\left(X,C\left(ℑ\right),A\right)$ where Y∈ℑ−{∅}. Let Z∈ℑ−{∅} with Z⊆Y. Then we have $C_{Z}\overset{˜}{\subseteq }C_{Y}$ with $C_{Z}\in C\left(ℑ\right)-\left\{0_{A}\right\}$ and so $C_{Z}=C_{Y}$. Thus, Z=Y and hence Y∈min⁡(X,ℑ). Conversely, let Y∈min⁡(X,ℑ). Then $C_{Y}\in$ $C\left(ℑ\right)-\left\{0_{A}\right\}$. Let $C_{Z}\in C\left(ℑ\right)-\left\{0_{A}\right\}$ where Z∈ℑ and $C_{Z}\overset{˜}{\subseteq }C_{Y}$. So, we have Z⊆Y with Z∈ℑ−{∅} and hence Z=Y. It follows that $C_{Z}=C_{Y}$ and hence $C_{Y}\in \min\left(X,C\left(ℑ\right),A\right)$. □

Theorem 4.15 *If* (X,τ,A) *is a STS with* τ⊆CSS(X,A)*, then* $\min\left(X,D\left(\tau \right)\right)=\left\{Y:C_{Y}\in \min\left(X,\tau ,A\right)\right\}$*.*

Proof Similar to the proof of Theorem 4.14. □

## 5 Soft homogeneity

In this section, we introduce and investigate soft homogeneity in soft topological spaces; in particular, we study the relation between soft homogeneity of a soft topological space and some ordinary topological spaces generated by this soft topological space, we study the relation between homogeneity of an ordinary topological space and some soft topological spaces generated by this ordinary topological space, and we introduce some properties of homogeneous soft topological spaces that contains a minimal soft open set.

Definition 5.1 A STS (X,τ,A) is called soft homogeneous if for any $a_{x},b_{y}\in SP\left(X,A\right)$, there exists a soft homeomorphism $f_{pu}:\left(X,\tau ,A\right)⟶\left(X,\tau ,A\right)$ such that $f_{pu}\left(a_{x}\right)=b_{y}$.

Theorem 5.2 *Let* (X,τ,A) *be a STS. Then the following are equivalent:*

*(a)* (X,τ,A) *is soft homogeneous.*

*(b) For any* $a_{x},b_{y}\in SP\left(X,A\right)$*, there is a soft homeomorphism* $f_{pu}:\left(X,\tau ,A\right)⟶\left(X,\tau ,A\right)$ *such that* p(x)=y *and* u(a)=b*.*

*(c) For any two pairs* (x,a),(y,b)∈X×A*, there is a soft homeomorphism* $f_{pu}:\left(X,\tau ,A\right)⟶\left(X,\tau ,A\right)$ *such that* p(x)=y *and* u(a)=b*.*

Proof Follows directly from the definition. □

Example 5.3 Let X={1,2,3,4}, A={a,b}, F={(a,{1,2}),(b,{3,4})}, G={(a,{3,4}),(b,{1,2})} and $\tau =\left\{0_{A},1_{A},F,G\right\}$. Then (X,τ,A) is soft homogeneous.

Proof Let $c_{x},d_{y}\in SP\left(X,A\right)=\left\{a_{1},a_{2},a_{3},a_{4},b_{1},b_{2},b_{3},b_{4}\right\}$.

**Case 1.** $c_{x}=a_{1}$ and $d_{y}=a_{2}$. Take u={(a,a),(b,b)} and p={(1,2),(2,1),(3,3),(4,4)}. Then $f_{pu}$ is a bijection with $f_{pu}\left(c_{x}\right)=d_{y}$.

$f_{pu}:\left(X,\tau ,A\right)⟶\left(X,\tau ,A\right)$ is soft open: $f_{pu}\left(F\right)=F$ and $f_{pu}\left(G\right)=G$.

$f_{pu}:\left(X,\tau ,A\right)⟶\left(X,\tau ,A\right)$ is soft continuous: $f_{pu}^{-1}=f_{p^{-1}u^{-1}}=f_{pu}$.

**Case 2**. $c_{x}=a_{1}$ and $d_{y}=a_{3}$. Take u={(a,a),(b,b)} and p={(1,3),(2,4),(3,1),(4,2)}. Then $f_{pu}$ is a bijection with $f_{pu}\left(c_{x}\right)=d_{y}$.

$f_{pu}:\left(X,\tau ,A\right)⟶\left(X,\tau ,A\right)$ is soft open: $f_{pu}\left(F\right)=G$ and $f_{pu}\left(G\right)=F$.

$f_{pu}:\left(X,\tau ,A\right)⟶\left(X,\tau ,A\right)$ is soft continuous: $f_{pu}^{-1}=f_{p^{-1}u^{-1}}=f_{pu}$.

**Case 3**. $c_{x}=a_{1}$ and $d_{y}=b_{1}$. Take u={(a,b),(b,a)} and p={(1,1),(2,2),(3,3),(4,4)}. Then $f_{pu}$ is a bijection with $f_{pu}\left(c_{x}\right)=d_{y}$.

$f_{pu}:\left(X,\tau ,A\right)⟶\left(X,\tau ,A\right)$ is soft open: $f_{pu}\left(F\right)=G$ and $f_{pu}\left(G\right)=F$.

$f_{pu}:\left(X,\tau ,A\right)⟶\left(X,\tau ,A\right)$ is soft continuous: $f_{pu}^{-1}=f_{p^{-1}u^{-1}}=f_{pu}$.

**Case 4**. $c_{x}=a_{1}$ and $d_{y}=b_{2}$. Take u={(a,b),(b,a)} and p={(1,2),(2,1),(3,3),(4,4)}. Then $f_{pu}$ is a bijection with $f_{pu}\left(c_{x}\right)=d_{y}$.

$f_{pu}:\left(X,\tau ,A\right)⟶\left(X,\tau ,A\right)$ is soft open: $f_{pu}\left(F\right)=G$ and $f_{pu}\left(G\right)=F$.

$f_{pu}:\left(X,\tau ,A\right)⟶\left(X,\tau ,A\right)$ is soft continuous: $f_{pu}^{-1}=f_{p^{-1}u^{-1}}=f_{pu}$.

**Case 5**. $c_{x}=a_{1}$ and $d_{y}=b_{3}$. Take u={(a,b),(b,a)} and p={(1,3),(2,4),(3,1),(4,2)}. Then $f_{pu}$ is a bijection with $f_{pu}\left(c_{x}\right)=d_{y}$.

$f_{pu}:\left(X,\tau ,A\right)⟶\left(X,\tau ,A\right)$ is soft open: $f_{pu}\left(F\right)=F$ and $f_{pu}\left(G\right)=G$.

$f_{pu}:\left(X,\tau ,A\right)⟶\left(X,\tau ,A\right)$ is soft continuous: $f_{pu}^{-1}=f_{p^{-1}u^{-1}}=f_{pu}$.

**Case 6**. $c_{x}=a_{1}$ and $d_{y}=b_{4}$. Take u={(a,b),(b,a)} and p={(1,4),(2,3),(3,2),(4,1)}. Then $f_{pu}$ is a bijection with $f_{pu}\left(c_{x}\right)=d_{y}$.

$f_{pu}:\left(X,\tau ,A\right)⟶\left(X,\tau ,A\right)$ is soft open: $f_{pu}\left(F\right)=F$ and $f_{pu}\left(G\right)=G$.

$f_{pu}:\left(X,\tau ,A\right)⟶\left(X,\tau ,A\right)$ is soft continuous: $f_{pu}^{-1}=f_{p^{-1}u^{-1}}=f_{pu}$. □

Each of the rest cases is similar to one of the above six cases.

Example 5.4 Let X={1,2,3,4} and A={a,b}. Let

F={(a,{1,2}),(b,∅)},G={(a,{3,4}),(b,∅)},H={(a,∅),(b,{1,2})},K={(a,∅),(b,{3,4})},

and B={F,G,H,K}. The soft topological space (X,τ,A) which has B as a soft base is soft homogeneous.

Proof Let $c_{x},d_{y}\in SP\left(X,A\right)=\left\{a_{1},a_{2},a_{3},a_{4},b_{1},b_{2},b_{3},b_{4}\right\}$.

**Case 1.** $c_{x}=a_{1}$ and $d_{y}=a_{2}$. Take u={(a,a),(b,b)} and p={(1,2),(2,1),(3,3),(4,4)}. Then $f_{pu}$ is a bijection with $f_{pu}\left(c_{x}\right)=d_{y}$.

$f_{pu}:\left(X,\tau ,A\right)⟶\left(X,\tau ,A\right)$ is soft open: $f_{pu}\left(F\right)=F$, $f_{pu}\left(G\right)=G$, $f_{pu}\left(H\right)=H$ and $f_{pu}\left(K\right)=K$.

$f_{pu}:\left(X,\tau ,A\right)⟶\left(X,\tau ,A\right)$ is soft continuous: $f_{pu}^{-1}=f_{p^{-1}u^{-1}}=f_{pu}$.

**Case 2.** $c_{x}=a_{1}$ and $d_{y}=a_{3}$. Take u={(a,a),(b,b)} and p={(1,3),(2,4),(3,1),(4,2)}. Then $f_{pu}$ is a bijection with $f_{pu}\left(c_{x}\right)=d_{y}$.

$f_{pu}:\left(X,\tau ,A\right)⟶\left(X,\tau ,A\right)$ is soft open: $f_{pu}\left(F\right)=G$, $f_{pu}\left(G\right)=F$, $f_{pu}\left(H\right)=K$ and $f_{pu}\left(K\right)=H$.

$f_{pu}:\left(X,\tau ,A\right)⟶\left(X,\tau ,A\right)$ is soft continuous: $f_{pu}^{-1}=f_{p^{-1}u^{-1}}=f_{pu}$.

**Case 3.** $c_{x}=a_{1}$ and $d_{y}=b_{1}$. Take u={(a,b),(b,a)} and p={(1,1),(2,2),(3,3),(4,4)}. Then $f_{pu}$ is a bijection with $f_{pu}\left(c_{x}\right)=d_{y}$.

$f_{pu}:\left(X,\tau ,A\right)⟶\left(X,\tau ,A\right)$ is soft open: $f_{pu}\left(F\right)=H$, $f_{pu}\left(G\right)=K$, $f_{pu}\left(H\right)=F$ and $f_{pu}\left(K\right)=G$.

$f_{pu}:\left(X,\tau ,A\right)⟶\left(X,\tau ,A\right)$ is soft continuous: $f_{pu}^{-1}=f_{p^{-1}u^{-1}}=f_{pu}$.

**Case 4.** $c_{x}=a_{1}$ and $d_{y}=b_{2}$. Take u={(a,b),(b,a)} and p={(1,2),(2,1),(3,3),(4,4)}. Then $f_{pu}$ is a bijection with $f_{pu}\left(c_{x}\right)=d_{y}$.

$f_{pu}:\left(X,\tau ,A\right)⟶\left(X,\tau ,A\right)$ is soft open: $f_{pu}\left(F\right)=H$, $f_{pu}\left(G\right)=K$, $f_{pu}\left(H\right)=F$ and $f_{pu}\left(K\right)=G$.

$f_{pu}:\left(X,\tau ,A\right)⟶\left(X,\tau ,A\right)$ is soft continuous: $f_{pu}^{-1}=f_{p^{-1}u^{-1}}=f_{pu}$.

**Case 5.** $c_{x}=a_{1}$ and $d_{y}=b_{3}$. Take u={(a,b),(b,a)} and p={(1,3),(2,4),(3,1),(4,2)}. Then $f_{pu}$ is a bijection with $f_{pu}\left(c_{x}\right)=d_{y}$.

$f_{pu}:\left(X,\tau ,A\right)⟶\left(X,\tau ,A\right)$ is soft open: $f_{pu}\left(F\right)=K$, $f_{pu}\left(G\right)=H$, $f_{pu}\left(H\right)=G$ and $f_{pu}\left(K\right)=F$.

$f_{pu}:\left(X,\tau ,A\right)⟶\left(X,\tau ,A\right)$ is soft continuous: $f_{pu}^{-1}=f_{p^{-1}u^{-1}}=f_{pu}$. □

Each of the rest cases is similar to one of the above five cases.

Example 5.5 Let X={1,2,3,4,5,6} and A={a,b,c}. Let

F={(a,{1,2}),(b,{3,4}),(c,{5,6})},G={(a,{3,4}),(b,{5,6}),(c,{1,2})},H={(a,{5,6}),(b,{1,2}),(c,{3,4})},

and B={F,G,H}. The soft topological space (X,τ,A) which has B as a soft base is soft homogeneous.

Proof Let $c_{x},d_{y}\in SP\left(X,A\right)=\{a_{1},a_{2},a_{3},a_{4},a_{5},a_{6},b_{1},b_{2},b_{3},b_{4},b_{5},b_{6},c_{1},c_{2},c_{3},c_{4},c_{5},c_{6}\}$.

**Case 1.** $c_{x}=a_{1}$ and $d_{y}=a_{2}$. Take u={(a,a),(b,b),(c,c)} and p={(1,2),(2,1),(3,3),(4,4),(5,5),(6,6)}. Then $f_{pu}$ is a bijection with $f_{pu}\left(c_{x}\right)=d_{y}$.

$f_{pu}:\left(X,\tau ,A\right)⟶\left(X,\tau ,A\right)$ is soft open: $f_{pu}\left(F\right)=F$, $f_{pu}\left(G\right)=G$, $f_{pu}\left(H\right)=H$.

$f_{pu}:\left(X,\tau ,A\right)⟶\left(X,\tau ,A\right)$ is soft continuous: $f_{pu}^{-1}=f_{p^{-1}u^{-1}}=f_{pu}$.

**Case 2.** $c_{x}=a_{1}$ and $d_{y}=a_{3}$. Take u={(a,a),(b,b),(c,c)} and p={(1,3),(2,4),(3,5),(4,6),(5,1),(6,2)}. Then $f_{pu}$ is a bijection with $f_{pu}\left(c_{x}\right)=d_{y}$.

$f_{pu}:\left(X,\tau ,A\right)⟶\left(X,\tau ,A\right)$ is soft open: $f_{pu}\left(F\right)=G$, $f_{pu}\left(G\right)=H$, $f_{pu}\left(H\right)=F$.

$f_{pu}:\left(X,\tau ,A\right)⟶\left(X,\tau ,A\right)$ is soft continuous: $f_{pu}^{-1}\left(F\right)=H$, $f_{pu}^{-1}\left(G\right)=F$, $f_{pu}^{-1}\left(H\right)=G$.

**Case 3.** $c_{x}=a_{1}$ and $d_{y}=b_{1}$. Take u={(a,b),(b,a),(c,c)} and p={(1,1),(2,2),(3,5),(4,6),(5,3),(6,4)}. Then $f_{pu}$ is a bijection with $f_{pu}\left(c_{x}\right)=d_{y}$.

$f_{pu}:\left(X,\tau ,A\right)⟶\left(X,\tau ,A\right)$ is soft open: $f_{pu}\left(F\right)=H$, $f_{pu}\left(G\right)=G$, $f_{pu}\left(H\right)=F$.

$f_{pu}:\left(X,\tau ,A\right)⟶\left(X,\tau ,A\right)$ is soft continuous: $f_{pu}^{-1}=f_{p^{-1}u^{-1}}=f_{pu}$.

**Case 4.** $c_{x}=a_{1}$ and $d_{y}=b_{2}$. Take u={(a,b),(b,a),(c,c)} and p={(1,2),(2,1),(3,5),(4,6),(5,3),(6,4)}. Then $f_{pu}$ is a bijection with $f_{pu}\left(c_{x}\right)=d_{y}$.

$f_{pu}:\left(X,\tau ,A\right)⟶\left(X,\tau ,A\right)$ is soft open: $f_{pu}\left(F\right)=H$, $f_{pu}\left(G\right)=G$, $f_{pu}\left(H\right)=F$.

$f_{pu}:\left(X,\tau ,A\right)⟶\left(X,\tau ,A\right)$ is soft continuous: $f_{pu}^{-1}=f_{p^{-1}u^{-1}}=f_{pu}$.

**Case 5.** $c_{x}=a_{1}$ and $d_{y}=b_{3}$. Take u={(a,b),(b,a),(c,c)} and p={(1,3),(2,4),(3,1),(4,2),(5,5),(6,6)}. Then $f_{pu}$ is a bijection with $f_{pu}\left(c_{x}\right)=d_{y}$.

$f_{pu}:\left(X,\tau ,A\right)⟶\left(X,\tau ,A\right)$ is soft open: $f_{pu}\left(F\right)=F$, $f_{pu}\left(G\right)=H$, $f_{pu}\left(H\right)=F$.

$f_{pu}:\left(X,\tau ,A\right)⟶\left(X,\tau ,A\right)$ is soft continuous: $f_{pu}^{-1}=f_{p^{-1}u^{-1}}=f_{pu}$.

**Case 6.** $c_{x}=a_{1}$ and $d_{y}=b_{5}$. Take u={(a,b),(b,a),(c,c)} and p={(1,5),(2,6),(3,3),(4,4),(5,1),(6,2)}. Then $f_{pu}$ is a bijection with $f_{pu}\left(c_{x}\right)=d_{y}$.

$f_{pu}:\left(X,\tau ,A\right)⟶\left(X,\tau ,A\right)$ is soft open: $f_{pu}\left(F\right)=G$, $f_{pu}\left(G\right)=F$, $f_{pu}\left(H\right)=G$.

$f_{pu}:\left(X,\tau ,A\right)⟶\left(X,\tau ,A\right)$ is soft continuous: $f_{pu}^{-1}=f_{p^{-1}u^{-1}}=f_{pu}$. □

Each of the rest cases is similar to one of the above six cases.

Theorem 5.6 *For any non-empty set X and for any set of parameters A, the STS* (X,SS(X,A),A) *is soft homogeneous.*

Proof Let $a_{x},b_{y}\in SP\left(X,A\right)$. Define p:X⟶X and u:A⟶A as follows:

$$p\left(t\right)=\left\{ \begin{array}{cc} y\; & \text{if }t=x\\ x\; & \text{if }t=y\\ t\; & \text{if }t\neq x\text{ and if }t\neq y \end{array}\right.$$

and

$$u\left(e\right)=\left\{ \begin{array}{cc} b\; & \text{if }e=a\\ a\; & \text{if }e=b\\ e\; & \text{if }e\neq a\text{ and if }e\neq b \end{array}\right.\text{.}$$

Then $f_{pu}$ is a soft homeomorphism with $f_{pu}\left(a_{x}\right)=b_{y}$. □

Theorem 5.7 *Let* (X,τ,A) *be a homogeneous STS. If there is* $a_{x}\in SP\left(X,A\right)\cap \tau$*, then* τ=SS(X,A)*.*

Proof It is sufficient to see that SP(X,A)⊆τ. Let $b_{y}\in SP\left(X,A\right)$. Since (X,τ,A) is soft homogeneous, then there is a soft homeomorphism $f_{pu}:\left(X,\tau ,A\right)⟶\left(X,\tau ,A\right)$ such that $f_{pu}\left(a_{x}\right)=b_{y}$. Since $a_{x}\in \tau$, then $b_{y}\in \tau$. It follows that SP(X,A)⊆τ. □

Corollary 5.8 *Let* (X,τ,A) *be a STS such that there is* $a_{x}\in SP\left(X,A\right)\cap \tau$*. Then* (X,τ,A) *is soft homogeneous iff* τ=SS(X,A)*.*

Proof Follows directly from Theorems 5.6 and 5.7. □

Definition 5.9 Let *X* be a non-empty set and let *α* be a cardinal number. A partition M of *X* is called an *α*-partition of *X* if α=|M| for all M∈M.

Definition 5.10 Let (X,ℑ) be a topological space, M be a base of (X,ℑ) and let *α* be a cardinal number. Then M is called an *α*-partition base of (X,ℑ) if M is an *α*-partition of *X*.

Theorem 5.11 *[33]* *Let* (X,ℑ) *be a topological space which contains* N∈min⁡(X,ℑ)*. Then* (X,ℑ) *is homogeneous iff* (X,ℑ) *has an* |N|*-partition base.*

Theorem 5.12 *[18]* *Let* $f_{pu}:\left(X,\tau ,A\right)⟶\left(Y,\sigma ,B\right)$ *be soft continuous. Then for every* a∈A*,* $p:\left(X,\tau_{a}\right)⟶\left(Y,\sigma_{u\left(a\right)}\right)$ *is continuous.*

Corollary 5.13 *Let* $f_{pu}:\left(X,\tau ,A\right)⟶\left(Y,\sigma ,B\right)$ *be a soft homeomorphism. Then for every* a∈A*,* $p:\left(X,\tau_{a}\right)⟶\left(Y,\sigma_{u\left(a\right)}\right)$ *is a homeomorphism.*

Theorem 5.14 *If* (X,τ,A) *is soft homogeneous, then for all* a∈A*,* $\left(X,\tau_{a}\right)$ *is homogeneous.*

Proof Let a∈A. Let x,y∈X. Since (X,τ,A) is soft homogeneous, then there is a soft homeomorphism $f_{pu}:\left(X,\tau ,A\right)⟶\left(X,\tau ,A\right)$ such that $f_{pu}\left(a_{x}\right)=a_{y}$. Then p(x)=y and u(a)=a. By Corollary 5.13, $p:\left(X,\tau_{a}\right)⟶\left(Y,\tau_{a}\right)$ is a homeomorphism with p(x)=y. It follows that $\left(X,\tau_{a}\right)$ is homogeneous. □

Theorem 5.15 *If* (X,τ,A) *is soft homogeneous, then for all* a,b∈A*,* $\left(X,\tau_{a}\right)$ *and* $\left(X,\tau_{b}\right)$ *are homeomorphic.*

Proof Let a,b∈A. Choose x∈X. Since (X,τ,A) is soft homogeneous, then there is a soft homeomorphism $f_{pu}:\left(X,\tau ,A\right)⟶\left(X,\tau ,A\right)$ such that $f_{pu}\left(a_{x}\right)=b_{x}$. Then u(a)=b. By Corollary 5.13, $p:\left(X,\tau_{a}\right)⟶\left(X,\tau_{b}\right)$ is a homeomorphism. It follows that $\left(X,\tau_{a}\right)$ and $\left(X,\tau_{b}\right)$ are homeomorphic. □

Each of the following two examples show that the converse of Theorem 5.14 is not true in general:

Example 5.16 Let X={1,2,3,4} and A={a,b}. Let

F={(a,{1}),(b,{1,2})},G={(a,{2}),(b,{1,2})},H={(a,{3}),(b,{3,4})},K={(a,{4}),(b,{3,4})},L={(a,∅),(b,{1,2})},M={(a,∅),(b,{3,4})},

$B=\left\{1_{A},F,G,H,K,L,M\right\}$, and let (X,τ,A) be the STS that has B as a soft base.

1. $\tau_{a}$ is the discrete topology and $\tau_{b}=\left\{\emptyset ,X,\left\{1,2\right\},\left\{3,4\right\}\right\}$, and by Theorem 5.11 both of $\left(X,\tau_{a}\right)$ and $\left(X,\tau_{b}\right)$ are homogeneous.

2. Since it is clear that $\left(X,\tau_{a}\right)$ and $\left(X,\tau_{b}\right)$ are not homeomorphic, then by Theorem 5.15, (X,τ,A) is not soft homogeneous.

Example 5.17 Let X=R and A={a,b}. Let ${ℑ}_{1}$ and ${ℑ}_{2}$ be the usual and the discrete topologies on R, respectively. Let $\tau =\{F\in SS\left(X,A\right):F\left(a\right)\in {ℑ}_{1}\text{ and }F\left(b\right)\in {ℑ}_{2}\}$.

1. $\tau_{a}={ℑ}_{1}$ and $\tau_{b}={ℑ}_{2}$. It is well known that both of $\left(X,\tau_{a}\right)$ and $\left(X,\tau_{b}\right)$ are homogeneous.

2. Since $\left(X,\tau_{a}\right)$ and $\left(X,\tau_{b}\right)$ are not homeomorphic, then by Theorem 5.15, (X,τ,A) is not soft homogeneous.

Lemma 5.18 *Let* $f_{pu}:\left(X,\tau ,A\right)⟶\left(Y,\sigma ,B\right)$ *be a soft mapping. Then for every* $C_{Z}\in CSS\left(Y,B\right)$*,* $f_{pu}^{-1}\left(C_{Z}\right)=C_{p^{-1}\left(Z\right)}$*.*

Proof Let $C_{Z}\in CSS\left(Y,B\right)$ and let a∈A. Then

$$\left(f_{pu}^{-1}\left(C_{Z}\right)\right)\left(a\right)=p^{-1}\left(C_{Z}\left(u\left(a\right)\right)\right)=p^{-1}\left(Z\right)\text{.}$$

Therefore, $f_{pu}^{-1}\left(C_{Z}\right)=C_{p^{-1}\left(Z\right)}$. □

Theorem 5.19 *Let* $f_{pu}:\left(X,\tau ,A\right)⟶\left(Y,\sigma ,B\right)$ *be a soft mapping with* τ⊆CSS(X,A) *and* σ⊆CSS(Y,B)*. Then* $f_{pu}$ *is soft continuous iff* p:(X,D(τ))⟶(Y,D(σ)) *is continuous.*

Proof *Necessity.* Suppose that $f_{pu}$ is soft continuous. Let Z∈D(σ). Then $C_{Z}\in \sigma$ and so $f_{pu}^{-1}\left(C_{Z}\right)\in \tau$. By Lemma 5.18, $f_{pu}^{-1}\left(C_{Z}\right)=C_{p^{-1}\left(Z\right)}$. Thus, $p^{-1}\left(Z\right)\in D\left(\tau \right)$. Hence, p:(X,D(τ))⟶(Y,D(σ)) is continuous.

*Sufficiency.* Suppose that p:(X,D(τ))⟶(Y,D(σ)) is continuous. Let $C_{Z}\in \sigma$. Then Z∈D(σ) and so $p^{-1}\left(Z\right)\in D\left(\tau \right)$. Thus, we have $C_{p^{-1}\left(Z\right)}\in \tau$. By Lemma 5.18, $C_{p^{-1}\left(Z\right)}=f_{pu}^{-1}\left(C_{Z}\right)$. Thus, $f_{pu}^{-1}\left(C_{Z}\right)\in \tau$. Therefore, $f_{pu}$ is soft continuous. □

Corollary 5.20 *Let* $f_{pu}:\left(X,\tau ,A\right)⟶\left(Y,\sigma ,B\right)$ *be a soft mapping with* τ⊆CSS(X,A) *and* σ⊆CSS(Y,B)*. Let* u:A⟶B *be a bijection. Then* $f_{pu}$ *is a soft homeomorphism iff* p:(X,D(τ))⟶(Y,D(σ)) *is a homeomorphism.*

Theorem 5.21 *Let* (X,τ,A) *be a STS with* τ⊆CSS(X,A)*. Then* (X,τ,A) *is soft homogeneous iff* (X,D(τ)) *is homogeneous.*

Proof *Necessity.* Suppose that (X,τ,A) is soft homogeneous. Let x,y∈X. Choose a∈A. Then there is a soft homeomorphism $f_{pu}:\left(X,\tau ,A\right)⟶\left(X,\tau ,A\right)$ such that $f_{pu}\left(a_{x}\right)=a_{y}$. So, p(x)=y. Also by Corollary 5.20, p:(X,D(τ))⟶(Y,D(τ)) is a homeomorphism with p(x)=y. Therefore, (X,D(τ)) is homogeneous.

*Sufficiency.* Suppose that (X,D(τ)) is homogeneous. Let $a_{x},b_{y}\in SP\left(X,A\right)$. Choose a homeomorphism p:(X,D(τ))⟶(X,D(τ)) such that p(x)=y. Choose a bijection u:A⟶A such that u(a)=b. By Corollary 5.20, $f_{pu}:\left(X,D\left(\tau \right),A\right)⟶\left(X,D\left(\tau \right),A\right)$ is a soft homeomorphism. Therefore, by Theorem 5.2, it follows that (X,D(τ),A) is soft homogeneous. □

Theorem 5.22 *Let* P:(X,ℑ)⟶(Y,ℜ) *be a function. Let* u:A⟶B *be a function between two sets of parameters A and B. Then* $f_{pu}:\left(X,C\left(ℑ\right),A\right)⟶\left(Y,C\left(ℜ\right),B\right)$ *is soft continuous iff p is continuous.*

Proof *Necessity.* Suppose that $f_{pu}:\left(X,C\left(ℑ\right),A\right)⟶\left(Y,C\left(ℜ\right),B\right)$ is soft continuous. Choose a∈A. Then by Theorem 5.12, $p:(X,{\left(C(ℑ)\right)}_{a})⟶\left(Y,{\left(C\left(ℜ\right)\right)}_{u\left(a\right)}\right)$ is continuous. By Theorem 3.17, ${\left(C\left(ℑ\right)\right)}_{a}=ℑ$ and ${\left(C\left(ℜ\right)\right)}_{u\left(a\right)}=ℜ$. It follows that P:(X,ℑ)⟶(Y,ℜ) is continuous.

*Sufficiency.* Suppose that P:(X,ℑ)⟶(Y,ℜ) is continuous. Let $C_{Z}\in C\left(ℜ\right)$. Then Z∈ℜ and so $p^{-1}\left(Z\right)\in ℑ$. Thus, we have $C_{p^{-1}\left(Z\right)}\in C\left(ℑ\right)$. By Lemma 5.18, $C_{p^{-1}\left(Z\right)}=f_{pu}^{-1}\left(C_{Z}\right)$. Thus, $f_{pu}^{-1}\left(C_{Z}\right)\in \tau$. Therefore, $f_{pu}$ is soft continuous. □

Corollary 5.23 *Let* P:(X,ℑ)⟶(Y,ℜ) *be a function. Let* u:A⟶B *be a bijection between two sets of parameters A and B. Then* $f_{pu}:\left(X,C\left(ℑ\right),A\right)⟶\left(Y,C\left(ℜ\right),B\right)$ *is a soft homeomorphism iff p is a homeomorphism.*

Theorem 5.24 *Let* (X,ℑ) *be a topological space and let A be a set of parameters. Then* (X,C(ℑ),A) *is soft homogeneous iff* (X,ℑ) *is homogeneous.*

Proof *Necessity.* Suppose that (X,C(ℑ),A) is soft homogeneous. Let x,y∈X. Choose a∈A. Then there is a soft homeomorphism $f_{pu}:\left(X,C\left(ℑ\right),A\right)⟶\left(X,C\left(ℑ\right),A\right)$ such that $f_{pu}\left(a_{x}\right)=a_{y}$. So, p(x)=y. Also by Corollary 5.23, p:(X,ℑ)⟶(X,ℑ) is a homeomorphism with p(x)=y. Therefore, (X,ℑ) is homogeneous.

*Sufficiency.* Suppose that (X,ℑ) is homogeneous. Let $a_{x},b_{y}\in SP\left(X,A\right)$. Choose a homeomorphism p:(X,ℑ)⟶(X,ℑ) such that p(x)=y. Choose a bijection u:A⟶A such that u(a)=b. By Corollary 5.23, $f_{pu}:\left(X,C\left(ℑ\right),A\right)⟶\left(X,C\left(ℑ\right),A\right)$ is a soft homeomorphism. Therefore, by Theorem 5.2, it follows that (X,C(ℑ),A) is soft homogeneous. □

Example 5.25 Consider the topological space $\left(R^{n},ℑ\right)$ where ℑ is the usual topology on $R^{n}$ and let A=R. It is well known that $\left(R^{n},ℑ\right)$ is homogeneous. Then by Theorem 5.24, $\left(R^{n},C\left(ℑ\right),A\right)$ is soft homogeneous.

Example 5.26 Consider the topological space ([0,1],ℑ) where ℑ is the usual topology on [0,1] and let A=N. It is well known that ([0,1],ℑ) is not homogeneous. Then by Theorem 5.24, ([0,1],C(ℑ),A) is not soft homogeneous.

Example 5.27 Consider the topological space $\left(S^{1},ℑ\right)$ where $S^{1}=\left\{\left(x,y\right):x,y\in R\text{ and }x^{2}+y^{2}=1\right\}$ and ℑ is the usual topology on $S^{1}$, and let A=Z. It is well known that $\left(S^{1},ℑ\right)$ is homogeneous. Then by Theorem 5.24, $\left(S^{1},C\left(ℑ\right),A\right)$ is soft homogeneous.

Example 5.28 Consider the topological space (X,ℑ) where $X=\left\{\left(x,y\right):x,y\in R\text{ and }x^{2}+y^{2}\leq 1\right\}$ and ℑ is the usual topology on *X*, and let A=Z. It is well known that (X,ℑ) is not homogeneous. Then by Theorem 5.24, (X,C(ℑ),A) is not soft homogeneous.

Theorem 5.29 *Let X be an initial universe and let A be a set of parameters. Let* $\left\{{ℑ}_{a}:a\in A\right\}$ *be an indexed family of topologies on X. If* $\left(X,\underset{a\in A}{⨁}{ℑ}_{a},A\right)$ *is soft homogeneous, then*

*(a)* $\left(X,{ℑ}_{b}\right)$ *is homogeneous for all* b∈A*.*

*(b)* $\left(X,{ℑ}_{b}\right)$ *is homeomorphic to* $\left(X,{ℑ}_{c}\right)$ *for all* b,c∈A*.*

Proof (a) Let b∈A. “Then by Theorem 5.14, $\left(X,{\left(\underset{a\in A}{⨁}{ℑ}_{a}\right)}_{b}\right)$ is homogeneous. By Theorem 3.7 we have ${\left(\underset{a\in A}{⨁}{ℑ}_{a}\right)}_{b}={ℑ}_{b}$ and hence $\left(X,{ℑ}_{b}\right)$ is homogeneous.

(b) Let a,b∈A. Then by Theorem 5.15, $\left(X,{\left(\underset{a\in A}{⨁}{ℑ}_{a}\right)}_{b}\right)$ and $\left(X,{\left(\underset{a\in A}{⨁}{ℑ}_{a}\right)}_{c}\right)$ are homeomorphic. By Theorem 3.7 we have ${\left(\underset{a\in A}{⨁}{ℑ}_{a}\right)}_{b}={ℑ}_{b}$ and ${\left(\underset{a\in A}{⨁}{ℑ}_{a}\right)}_{c}={ℑ}_{c}$. It follows that $\left(X,{ℑ}_{b}\right)$ is homeomorphic to $\left(X,{ℑ}_{c}\right)$. □

Question 5.30 *Let* $\left\{\left(X,{ℑ}_{a}\right):a\in A\right\}$ *be an indexed family of topological spaces such that*

*(a)* $\left(X,{ℑ}_{b}\right)$ *is homogeneous for all* b∈A*.*

*(b)* $\left(X,{ℑ}_{b}\right)$ *is homeomorphic to* $\left(X,{ℑ}_{c}\right)$ *for all* b,c∈A*.*

*Is it true that* $\left(X,\underset{a\in A}{⨁}{ℑ}_{a},A\right)$ *is soft homogeneous?*

Theorem 5.31 *Let* $p:\left(X,{ℑ}_{1}\right)⟶\left(Y,{ℑ}_{2}\right)$ *be a function between two topological spaces and let* u:A⟶B *be a function. Then* $f_{pu}:\left(X,\tau \left({ℑ}_{1}\right),A\right)⟶\left(Y,\tau \left({ℑ}_{2}\right),B\right)$ *is soft continuous iff* $p:\left(X,{ℑ}_{1}\right)⟶\left(Y,{ℑ}_{2}\right)$ *is continuous.*

Proof *Necessity.* Suppose that $f_{pu}:\left(X,\tau \left({ℑ}_{1}\right),A\right)⟶\left(Y,\tau \left({ℑ}_{2}\right),B\right)$ is soft continuous. Choose a∈A. Then by Theorem 5.12, $p:(X,{\left(\tau \left({ℑ}_{1}\right)\right)}_{a})⟶\left(Y,{\left(\tau \left({ℑ}_{2}\right)\right)}_{u\left(a\right)}\right)$ is continuous. By Corollary 3.8, ${\left(\tau \left({ℑ}_{1}\right)\right)}_{a}={ℑ}_{1}$ and ${\left(\tau \left({ℑ}_{2}\right)\right)}_{u\left(a\right)}={ℑ}_{2}$. Thus, $p:\left(X,{ℑ}_{1}\right)⟶\left(Y,{ℑ}_{2}\right)$ is continuous.

*Sufficiency.* Suppose that $p:\left(X,{ℑ}_{1}\right)⟶\left(Y,{ℑ}_{2}\right)$ is continuous. Let $G\in \tau \left({ℑ}_{2}\right)$ and let a∈A. Then $\left(f_{pu}^{-1}\left(G\right)\right)\left(a\right)=p^{-1}\left(G\left(u\left(a\right)\right)\right)$. Since u(a)∈B, then $G\left(u\left(a\right)\right)\in {ℑ}_{2}$. Since $p:\left(X,{ℑ}_{1}\right)⟶\left(Y,{ℑ}_{2}\right)$ is continuous, then $p^{-1}\left(G\left(u\left(a\right)\right)\right)\in {ℑ}_{1}$ and so $\left(f_{pu}^{-1}\left(G\right)\right)\left(a\right)\in {ℑ}_{1}$. Therefore, $\left(f_{pu}^{-1}\left(G\right)\right)\in \tau \left({ℑ}_{1}\right)$ and hence $f_{pu}:\left(X,\tau \left({ℑ}_{1}\right),A\right)⟶\left(Y,\tau \left({ℑ}_{2}\right),B\right)$ is soft continuous. □

Theorem 5.32 *Let* $p:\left(X,{ℑ}_{1}\right)⟶\left(Y,{ℑ}_{2}\right)$ *be a function between two topological spaces and let* u:A⟶B *be a bijection. Then* $f_{pu}:(X,\tau \left({ℑ}_{1}\right),A)⟶\left(Y,\tau \left({ℑ}_{2}\right),B\right)$ *is a soft homeomorphism iff* $p:\left(X,{ℑ}_{1}\right)⟶\left(Y,{ℑ}_{2}\right)$ *is a homeomorphism.*

Proof *Necessity.* Suppose that $f_{pu}:\left(X,\tau \left({ℑ}_{1}\right),A\right)⟶\left(Y,\tau \left({ℑ}_{2}\right),B\right)$ is a soft homeomorphism. Then p:X⟶Y is a bijection. By Theorem 5.31, $p:\left(X,{ℑ}_{1}\right)⟶\left(Y,{ℑ}_{2}\right)$ and $p^{-1}:\left(Y,{ℑ}_{2}\right)⟶\left(X,{ℑ}_{1}\right)$ are continuous. It follows that $p:\left(X,{ℑ}_{1}\right)⟶\left(Y,{ℑ}_{2}\right)$ is a homeomorphism.

*Sufficiency.* Suppose that $p:\left(X,{ℑ}_{1}\right)⟶\left(Y,{ℑ}_{2}\right)$ is a homeomorphism. Then p:X⟶Y is a bijection. Since we have *p* and *u* are bijections, then $f_{pu}:\left(X,\tau \left({ℑ}_{1}\right),A\right)⟶\left(Y,\tau \left({ℑ}_{2}\right),B\right)$ is a bijection. Since $p:\left(X,{ℑ}_{1}\right)⟶\left(Y,{ℑ}_{2}\right)$ is continuous, then by Theorem 5.31, $f_{pu}:\left(X,\tau \left({ℑ}_{1}\right),A\right)⟶\left(Y,\tau \left({ℑ}_{2}\right),B\right)$ is soft continuous. Since $p^{-1}:\left(Y,{ℑ}_{2}\right)⟶\left(X,{ℑ}_{1}\right)$ is continuous, then again by Theorem 5.31, $f_{p^{-1}u^{-1}}:\left(Y,\tau \left({ℑ}_{2}\right),B\right)⟶\left(X,\tau \left({ℑ}_{1}\right),A\right)$ is soft continuous. If follows that $f_{pu}:\left(X,\tau \left({ℑ}_{1}\right),A\right)⟶\left(Y,\tau \left({ℑ}_{2}\right),B\right)$ is a soft homeomorphism. □

The following result answers Question 5.30 partially:

Theorem 5.33 *Let* (X,ℑ) *be a topological space and let A be a set of parameters. Then* (X,τ(ℑ),A) *is soft homogeneous iff* (X,ℑ) *is homogeneous.*

Proof *Necessity.* Suppose that (X,τ(ℑ),A) is soft homogeneous. For all a∈A, put ${ℑ}_{a}=ℑ$. Then $\tau \left(ℑ\right)=\underset{a\in A}{⨁}{ℑ}_{a}$ and so $\left(X,\underset{a\in A}{⨁}{ℑ}_{a},A\right)$ is soft homogeneous. By Theorem 5.29 (a), it follows that (X,ℑ) is homogeneous.

*Sufficiency.* Suppose that (X,ℑ) is homogeneous. Let $a_{x},b_{y}\in SP\left(X,A\right)$. Choose a homeomorphism p:(X,ℑ)⟶(X,ℑ) such that p(x)=y. Choose a bijection u:A⟶A such that u(a)=b. By Theorem 5.32, $f_{pu}:\left(X,\tau \left(ℑ\right),A\right)⟶\left(X,\tau \left(ℑ\right),A\right)$ is a soft homeomorphism. Therefore, by Theorem 5.2, it follows that (X,τ(ℑ),A) is soft homogeneous. □

Example 5.34 Consider the topological space $\left(R^{n},ℑ\right)$ where ℑ is the usual topology on $R^{n}$ and let A=R. It is well known that $\left(R^{n},ℑ\right)$ is homogeneous. Then by Theorem 5.33, $\left(R^{n},\tau \left(ℑ\right),A\right)$ is soft homogeneous.

Example 5.35 Consider the topological space (X,ℑ) where *X* is the Cantor set and ℑ is the usual topology on *X*, and let A=Z. It is well known that (X,ℑ) is homogeneous. Then by Theorem 5.33, (X,τ(ℑ),A) is soft homogeneous.

For every F∈SS(X,A), the set Supp(F)={a∈A:F(a)≠∅} is called the support of *F*.

Theorem 5.36 *Let* $f_{pu}:\left(X,\tau ,A\right)⟶\left(Y,\sigma ,B\right)$ *be a soft mapping and let* F∈SS(X,A)*. Then* $Supp\left(f_{pu}\left(F\right)\right)=u\left(Supp\left(F\right)\right)$*.*

Proof To see that $Supp\left(f_{pu}\left(F\right)\right)\subseteq u\left(Supp\left(F\right)\right)$, let $b\in Supp\left(f_{pu}\left(F\right)\right)$. Then $\left(f_{pu}\left(F\right)\right)\left(b\right)\neq \emptyset$. Note that $\left(f_{pu}\left(F\right)\right)\left(b\right)=\cup \left\{p\left(F\left(a\right)\right):a\in u^{-1}\left(b\right)\right\}$. So, there is $a\in u^{-1}\left(b\right)$ such that p(F(a))≠∅. Thus, we have a∈Supp(F) and u(a)=b. Therefore, b∈u(Supp(F)). This ends the proof that $Supp\left(f_{pu}\left(F\right)\right)\subseteq u\left(Supp\left(F\right)\right)$. To see that $u\left(Supp\left(F\right)\right)\subseteq Supp\left(f_{pu}\left(F\right)\right)$, let b∈u(Supp(F)). Then there is a∈Supp(F) such that b=u(a). Thus, we have p(F(a))≠∅ and $p\left(F\left(a\right)\right)\subseteq \cup \left\{p\left(F\left(a\right)\right):a\in u^{-1}\left(b\right)\right\}=\left(f_{pu}\left(F\right)\right)\left(b\right)$. It follows that $b\in Supp\left(f_{pu}\left(F\right)\right)$. This ends the proof that $u\left(Supp\left(F\right)\right)\subseteq Supp\left(f_{pu}\left(F\right)\right)$. □

Theorem 5.37 *Let* $f_{pu}:\left(X,\tau ,A\right)⟶\left(Y,\sigma ,B\right)$ *be a soft mapping with* u:A⟶B *is an injection, and let* F∈SS(X,A)*. Then* $\left|Supp\left(f_{pu}\left(F\right)\right)\right|=\left|Supp\left(F\right)\right|$*.*

Proof By Theorem 5.36, $Supp\left(f_{pu}\left(F\right)\right)=u\left(Supp\left(F\right)\right)$ and so $\left|Supp\left(f_{pu}\left(F\right)\right)\right|=\left|u\left(Supp\left(F\right)\right)\right|$. Since u:A⟶B is an injection, then |u(Supp(F))|=|Supp(F)|. It follows that $\left|Supp\left(f_{pu}\left(F\right)\right)\right|=\left|Supp\left(F\right)\right|$. □

Theorem 5.38 *Let* (X,τ,A) *be a homogeneous STS which contains* F∈min⁡(X,τ,A)*. Then*

*(a)* min⁡(X,τ,A) *is a soft base for* (X,τ,A)*.*

*(b) For all* G∈min⁡(X,τ,A) *and for all* a,b∈A *with* F(a)≠∅ *and* G(b)≠∅*,* |F(a)|=|G(b)|*.*

*(c) For all* G∈min⁡(X,τ,A)*,* |Supp(F)|=|Supp(G)|*.*

Proof (a) We apply Proposition 2.29. Clearly that min⁡(X,τ,A)⊆τ. Let $G\in \tau -\left\{0_{A}\right\}$ and let $b_{y}\overset{˜}{\in }G$. Choose $a_{x}\overset{˜}{\in }F$. Since (X,τ,A) is soft homogeneous, then there is a soft homeomorphism $f_{pu}:\left(X,\tau ,A\right)⟶\left(X,\tau ,A\right)$ such that $f_{pu}\left(a_{x}\right)=b_{y}$. So, $b_{y}\overset{˜}{\in }f_{pu}\left(F\right)\overset{˜}{\cap }G$. Since F∈min⁡(X,τ,A), then by Theorem 4.4, $f_{pu}\left(F\right)\in \min\left(X,\tau ,A\right)$. Thus, by Proposition 4.2, $f_{pu}\left(F\right)\overset{˜}{\subseteq }G$. Therefore, min⁡(X,τ,A) is a soft base for (X,τ,A).

(b) Let G∈min⁡(X,τ,A) and let a,b∈A such that F(a)≠∅ and G(b)≠∅. Choose x∈F(a) and y∈G(b). Then $a_{x}\overset{˜}{\in }F$ and $b_{y}\overset{˜}{\in }G$. Since (X,τ,A) is soft homogeneous, then there is a soft homeomorphism $f_{pu}:\left(X,\tau ,A\right)⟶\left(X,\tau ,A\right)$ such that $f_{pu}\left(a_{x}\right)=b_{y}$. Since F∈min⁡(X,τ,A), by Theorem 4.4, $f_{pu}\left(F\right)\in \min\left(X,\tau ,A\right)$. Since $a_{x}\overset{˜}{\in }F$, then $b_{y}=f_{pu}\left(a_{x}\right)\overset{˜}{\in }f_{pu}\left(F\right)$. Since $f_{pu}\left(F\right),G\in \min\left(X,\tau ,A\right)$ and $b_{y}\;\overset{˜}{\in }G\overset{˜}{\cap }f_{pu}\left(F\right)$, then by Proposition 4.3, $f_{pu}\left(F\right)=G$. Note that $\left|G\left(b\right)\right|=\left|\left(f_{pu}\left(F\right)\right)\left(b\right)\right|=\left|p\left(F\left(u^{-1}\left(b\right)\right)\right)\right|=\left|p\left(F\left(a\right)\right)\right|$. Since *p* is bijective, then |p(F(a))|=|F(a)|. Therefore, |F(a)|=|G(b)|.

(c) Choose $a_{x}\overset{˜}{\in }F$ and $b_{y}\overset{˜}{\in }G$. Since (X,τ,A) is soft homogeneous, then there is a soft homeomorphism $f_{pu}:\left(X,\tau ,A\right)⟶\left(X,\tau ,A\right)$ such that $f_{pu}\left(a_{x}\right)=b_{y}$. By Theorem 4.4, $f_{pu}\left(F\right)\in \min\left(X,\tau ,A\right)$. Since $b_{y}\overset{˜}{\in }G\overset{˜}{\cap }f_{pu}\left(F\right)$, then by Proposition 4.3, $f_{pu}\left(F\right)=G$. Thus, by Theorem 5.37, it follows that $\left|Supp\left(F\right)\right|=\left|Supp\left(f_{pu}\left(F\right)\right)\right|=\left|Supp\left(G\right)\right|$. □

Corollary 5.39 *Let* (X,τ,A) *be a homogeneous STS with the property that* Supp(G)=A *for all* $G\in \tau -\left\{0_{A}\right\}$*. If* F∈min⁡(X,τ,A)*, then we have the following:*

*(a)* min⁡(X,τ,A) *is a soft base for* (X,τ,A)*.*

*(b) For all* G∈min⁡(X,τ,A) *and for all* a,b∈A*,* |F(a)|=|G(b)|*.*

## 6 Conclusion

In this paper, homogeneity as an ordinary topological property is extended to include soft topological spaces. Also, the study of soft minimal open sets is continued. The results deals mainly with the relation between the generated soft topology and the given indexed family of topologies defined in [17]. Also, some properties of soft homogeneous soft topological spaces that contains a minimal soft open set are given. In our future study, the following topics could be considered: 1) To define soft homogeneity components; 2) To extend countable dense homogeneity to include soft topological spaces.

## Declarations

### Author contribution statement

Samer Al Ghour: Conceived and designed the experiments; Analyzed and interpreted the data; contributed reagents, materials, analysis tools or data; Wrote the paper.

Awatef Bin-Saadon: Performed the experiments; Analyzed and interpreted the data; contributed reagents, materials, analysis tools or data; Wrote the paper.

### Funding statement

This research did not receive any specific grant from funding agencies in the public, commercial, or not-for-profit sectors.

### Competing interest statement

The authors declare no conflict of interest.

### Additional information

No additional information is available for this paper.
